# Cross-sectional analysis of the correlation between serum uric acid and trabecular bone score: NHANES 2005–2008

**DOI:** 10.1038/s41598-023-48739-5

**Published:** 2023-12-06

**Authors:** Yanlei Li, Jinxin Tan, Jinlong Tian, Jiongnan Xu, Haiyu Shao, Jun Zhang, Tingxiao Zhao, Yazeng Huang

**Affiliations:** 1 Center for Plastic & Reconstructive Surgery, Department of Orthopedics, Zhejiang Provincial People’s Hospital (Affiliated People’s Hospital, Hangzhou Medical College), Shangtang Road 158#, Hangzhou, 310014 Zhejiang China; 2 Department of Orthopedics, Zhejiang Medical & Health Group Hangzhou Hospital, Hangzhou, Zhejiang China

**Keywords:** Endocrine system and metabolic diseases, Medical research

## Abstract

Serum uric acid (SUA) has been discovered to be associated with bone mineral density (BMD), but its relationship with trabecular bone score (TBS) remains unclear. Thus, the aim of our study was to investigate the association between SUA levels and TBS. Our study included 5895 individuals over 20 years old (3061 men and 2834 women) from NHANES 2005–2008. To analyze the association between SUA and TBS, multivariate linear regression models with covariate adjustments were applied. Furthermore, population description, stratified analysis, single factor analysis, smooth curve fitting, interaction analysis, and threshold effect and saturation effect analysis were also conducted. After adjusting for covariates, SUA showed a strong negative relationship with total TBS (β = 0.319; 95% CI 0.145–0.494; P < 0.001). The relationship between SUA levels and total TBS was found to be nonlinear, with inflection points at 4.8 mg/dL for the overall population, 4.2 mg/dL for women, and 5.7 mg/dL for non-Hispanic whites, indicating a saturation effect. Additionally, no interactions were found in any of the subgroups. Our study found a negative association between SUA and total TBS in adults. Maintaining SUA at a saturated level can benefit in preventing osteoporosis and fractures.

## Introduction

Osteoporosis is a chronic systemic bone disease characterized by reduced BMD and destruction of bone structure, which seriously affects physical and mental health^1^. According to estimates based on US Census data, in 2010, there were 10.2 million elderly individuals affected by osteoporosis, and an additional 43.4 million elderly individuals had low bone density, which placed them in a high-risk category for osteoporotic fractures^2^. The BMD measurement is commonly used to diagnose osteoporosis. Although BMD can identify many individuals who are at risk, using BMD alone to assess fracture risk may not be sufficient, especially for people with normal or higher BMD levels^3^. TBS is a novel method to reflect bone quality based on the gray-level texture parameters of the lumbar DXA images^4^. Compared with BMD, TBS helps detect populations with degraded bone microstructure but normal BMD, and it more accurately predicts osteoporotic fractures^5,6^. Therefore, TBS, which can reflect bone microstructure and evaluate bone quality, has attracted much attention.

SUA is the end product of purine metabolism in humans. Elevated SUA levels or hyperuricemia can lead to a series of diseases, such as gouty arthritis, diabetes mellitus (DM), chronic kidney disease (CKD), cardiovascular disease, and metabolic syndrome^7–10^. Meanwhile, recent studies have found that increased SUA levels may be linked to a decreased prevalence of osteoporosis^11^. Its mechanism may be related to the antioxidant effect of SUA^12^. Furthermore, SUA can also affect the level of bone metabolism by regulating the activities of osteoclasts and osteoblasts^13,14^. However, some studies have not supported the association between uric acid and bone density or osteoporosis^15–17^. Therefore, the effect of SUA remains controversial.

Currently, the relationship between SUA and TBS is seldom reported. To address this, we collected data from the National Health and Nutrition Examination Survey (NHANES) from 2005 to 2008, through strict inclusion criteria and covariate adjustments, to clarify the relationship between SUA and total TBS.

## Method

**Figure 1:**
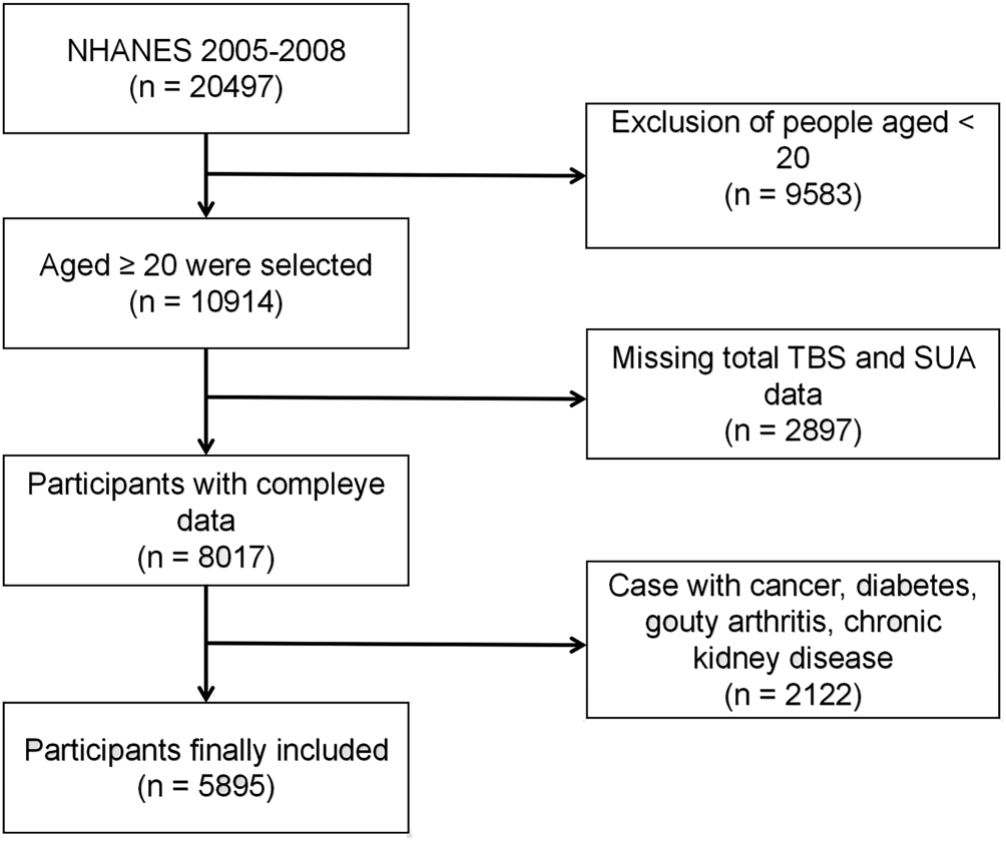
Flowchart of participants selection.

NHANES is a nationally representative cross-sectional survey aimed at providing abundant information on the overall health and nutritional status of the United States (US) population^18^. Detailed data on NHANES can be found on the Internet and has been approved by the National Center for Health Statistics (NCHS) Ethics Review Board^19^. For this cross-sectional study, we collected 20,974 individuals from NHANES 2005–2008 and ultimately identified 5895 participants through strict inclusion criteria. The specific process is shown in Fig. 1. Exclusion criteria included: age < 20 years old; missing SUA and total TBS data; diabetes mellitus; rheumatoid arthritis; chronic kidney disease (estimated glomerular filtration rate [eGFR < 60 mL/min/1.73 m^2^]); cancer; or participants using diphosphonate, glucocorticoids, estrogen, diuretics, allopurinol therapy. Finally, out of 20,497 participants, a total of 5895 eligible individuals without serious illnesses were included in the study.

The study measured the independent variable, SUA, using the Beckman Synchron LX20. The dependent variable was total TBS. It was considered to be a textural index and measured by evaluating gray-level variations of lumbar spine DXA scanning pixels. The spine scans were obtained using Hologic QDR-4500A fan-beam densitometers (Hologic, Inc., Bedford, Massachusetts). Subsequently, the total TBS score was estimated in adults aged 20 years and older through TBS software (Med-Imap SA TBS Calculator version 2.1.0.2). Meanwhile, for statistical analysis, quartiles of SUA levels were divided into four groups: the first quartile (Q1): 0.5–4.4 mg/dL, the second quartile (Q2): 4.5–5.3 mg/dL, the third quartile (Q3): 5.4–6.2 mg/dL, and the fourth quartile (Q4): 6.3–11 mg/dL.

Based on prior research and clinical experience, we incorporated the subsequent covariates that might influence the association between SUA and TBS. The continuous covariates in this study were age, height, weight, body mass index (BMI), waist circumference, alanine transaminase (ALT), alkaline phosphatase (ALP), aspartate aminotransferase (AST), total protein, cholesterol, high-density lipoprotein cholesterol (HDL-C), serum phosphorus, blood urea nitrogen, serum calcium, serum albumin, serum creatinine, C-reactive protein (CRP), total femur bone mineral density (BMD), total spine bone mineral density (BMD), calcium supplementation, and estimated glomerular filtration rate (eGFR). The categorical variables among the covariates were gender, age, BMI, race/ethnicity, education level, marital status, income to poverty ratio, drinking status (Had at least 12 alcohol drinks a year?), sleep, smoke. Details of each variable are available on the NHANES website. The poverty income ratio is determined by dividing family income by the poverty guidelines set by the Department of Health and Human Services (HHS). This ratio is used to measure poverty and is categorized as "mild" (poverty income ratio < 1.99), "moderate" (1.99 ≤ poverty income ratio ≤ 3.49), or "severe" (poverty income ratio > 3.49). The equation for estimating GFR^20^ is shown in Supplementary Table S1.

### Statistical analyses

Before conducting the data analysis for this study, we performed normality tests on all variables using histograms and P-P plots. The comparison of continuous variables was done using a weighted linear regression model, while the comparison of categorical variables was done using a weighted chi-square test. The final analysis was expressed as mean ± standard deviation (SD; continuous variable) or percentages (categorical variable). In three different models, weighted multivariate linear regression analysis was used to evaluate the correlation between SUA and total TBS. The three models are as follows: Model 1, a non-adjusted model; Model 2, minimally adjusted for gender, age, and race/ethnicity; Model 3, fully adjusted for all covariates. We also conducted group analysis and performed interaction tests on subgroups. Furthermore, the generalized additive models (GAM) and smooth curve fittings were further applied in the fully adjusted model to check the nonlinear or linear correlation between SUA and total TBS. Determine whether there is a non-linear relationship based on the log-likelihood ratio. If there is a non-linear relationship, a two-stage linear regression model is used to calculate the inflection point of SUA on total TBS using a recursive algorithm.

When the p-value is less than 0.05 (two-sided), it has statistical significance. All statistical analyses were performed using EmpowerStats (http://www.empowerstats.com, X&Y Solutions, Inc, Boston, MA) and R statistics packages (http://www.R-project.org, The R Foundation).

### Ethics statement

According to local laws and institutional requirements, this study did not require ethical review and approval. Participants provided written informed consent to participate in this study.

## Results

### Characteristics of the study population

The study involved a total of 5895 participants aged 20 years or older, with an average age of 44.66 ± 16.00 years. Weighted demographics and clinical characteristics according to the quartile of SUA were shown in Table 1. The missing data for covariates are shown in Supplementary Table S2. Missing data for categorical variables were treated as "Not recorded" categories; missing data for continuous variables were coded as mean values. There were significant differences in baseline characteristics of the SUA quartiles except for income to poverty ratio and race/ethnicity. Men, more than high school, married/living with partner, drinkers, and sleep time (7–8 h) had higher levels of SUA in the top quartile (Q4). Interestingly, women may have lower levels of SUA in the quartiles (Q1 and Q2).

**Table 1:** Characteristics of study participants based on SUA quartile, weighted.

Characteristic	Serum uric acid (mg/dL)					
	Total	Q1 (0.5–4.4)	Q2 (4.4–5.3)	Q3 (5.3–6.2)	Q4 (6.2–11)	P-value
N	5895	1417	1490	1411	1577	
Age (years)	42.803 ± 14.472	41.84 ± 14.65	42.88 ± 14.48	43.28 ± 14.47	43.19 ± 14.27	0.0281
Weight (kg)	79.903 ± 18.136	68.62 ± 14.86	75.93 ± 15.93	83.98 ± 17.00	90.08 ± 16.80	< 0.0001
Height (cm)	169.562 ± 9.906	164.29 ± 7.90	167.58 ± 9.48	171.71 ± 10.21	174.22 ± 8.83	< 0.0001
BMI (kg/m^2^)	27.717 ± 5.613	25.39 ± 5.14	27.05 ± 5.38	28.51 ± 5.54	29.71 ± 5.42	< 0.0001
Waist circumference (cm)	95.442 ± 14.425	87.35 ± 12.73	92.65 ± 13.30	98.27 ± 13.48	102.78 ± 13.19	< 0.0001
Calcium supplementation (mg)	930.341 ± 552.087	901.94 ± 535.53	922.57 ± 532.66	933.40 ± 567.25	960.28 ± 568.76	0.0314
Serum creatinine (mg/dL)	0.868 ± 0.171	0.76 ± 0.13	0.83 ± 0.15	0.91 ± 0.16	0.97 ± 0.16	< 0.0001
eGFR (mL/min/1.73 m^2^)	97.444 ± 18.027	102.11 ± 17.49	97.90 ± 18.18	96.29 ± 17.98	93.85 ± 17.46	< 0.0001
Serum albumin (g/L)	42.941 ± 3.063	42.28 ± 3.00	42.71 ± 3.03	43.04 ± 3.03	43.67 ± 3.02	< 0.0001
Alanine aminotransferase (U/L)	26.387 ± 18.451	20.44 ± 17.55	24.39 ± 16.07	27.71 ± 16.50	32.38 ± 20.76	< 0.0001
Aspartate aminotransferase (U/L)	25.684 ± 14.006	22.81 ± 18.06	24.67 ± 11.18	26.52 ± 11.95	28.46 ± 13.17	< 0.0001
Alkaline phosphatase (U/L)	66.558 ± 21.283	61.47 ± 19.97	66.24 ± 20.76	68.82 ± 22.43	69.44 ± 21.01	< 0.0001
Blood urea nitrogen (mg/dL)	12.017 ± 3.810	11.04 ± 3.46	11.77 ± 3.75	12.21 ± 3.72	12.95 ± 4.01	< 0.0001
Serum calcium (mg/dL)	9.460 ± 0.351	9.40 ± 0.36	9.45 ± 0.35	9.47 ± 0.33	9.52 ± 0.35	< 0.0001
Cholesterol (mg/dL)	200.399 ± 39.926	194.49 ± 37.01	200.94 ± 41.91	201.16 ± 38.90	204.56 ± 40.87	< 0.0001
Serum phosphorus (mg/dL)	3.781 ± 0.561	3.84 ± 0.52	3.80 ± 0.57	3.75 ± 0.56	3.74 ± 0.59	< 0.0001
Total protein (g/L)	71.388 ± 4.315	70.57 ± 4.26	70.96 ± 4.14	71.63 ± 4.32	72.30 ± 4.32	< 0.0001
C-reactive protein (mg/L)	0.346 ± 0.736	0.29 ± 0.56	0.33 ± 0.55	0.38 ± 0.91	0.38 ± 0.84	0.0009
HDL-C (mmol/L)	1.377 ± 0.410	1.55 ± 0.42	1.43 ± 0.41	1.32 ± 0.39	1.23 ± 0.35	< 0.0001
Total femur BMD(g/cm^2^)	0.988 ± 0.146	0.92 ± 0.14	0.97 ± 0.14	1.01 ± 0.14	1.04 ± 0.13	< 0.0001
Total spine BMD (g/cm^2^)	1.042 ± 0.130	1.02 ± 0.13	1.04 ± 0.14	1.05 ± 0.13	1.06 ± 0.12	< 0.0001
Total TBS	1.402 ± 0.135	1.44 ± 0.11	1.42 ± 0.12	1.39 ± 0.14	1.36 ± 0.15	< 0.0001
Gender (%)						< 0.0001
Men	50.772	13.35	37.05	66.31	83.24	
Women	49.228	86.65	62.95	33.69	16.76	
Income to poverty ratio (%)						0.247
Low	39.83	40.226	42.349	39.05	37.793	
Middle	26.751	27.17	25.034	26.931	27.838	
High	33.418	32.604	32.617	34.018	34.369	
Race/ethnicity (%)						0.7084
Mexican American	9.003	9.19	9.79	8.87	8.25	
Other race/ethnicity	10.328	11.13	9.42	11.06	9.79	
Non-Hispanic white	70.578	69.51	70.52	69.98	72.12	
Non-Hispanic black	10.09	10.18	10.27	10.1	9.84	
Education (%)						0.0322
Less than high school	16.949	15.32	17.7	18.41	16.45	
High school	24.073	22.04	26.09	24.14	24.02	
More than high school	58.928	62.56	56.21	57.45	59.41	
Not recorded	0.051	0.08			0.11	
Marital status (%)						0.0014
Married/living with a partner	66.487	66.71	63.49	66.25	69.21	
Widowed/divorced/separated	15.326	16.41	17.55	15.67	12.03	
Never married	18.138	16.81	18.82	18.08	18.77	
Not recorded	0.049	0.06	0.14			
Drinking status (%)						< 0.0001
Yes	75.186	66.11	72.25	78.41	83.19	
No	21.378	29.42	24.8	17.95	14.05	
Not recorded	3.436	4.48	2.95	3.64	2.76	
Smoke (%)						< 0.0001
Yes	25.536	22.54	25.93	28.76	25.04	
No	22.031	17.31	18.63	23.23	27.43	
Not recorded	52.433	60.15	55.45	47	47.53	
Sleep (%)						0.0003
< 6 h	13.367	11.29	13.72	13.51	14.8	
= 6 h	23.033	20.02	23.45	25.28	23.39	
7–8 h	57.741	61.02	57.26	56.17	56.61	
> 8 h	5.86	7.68	5.57	5.04	5.2	

Data are expressed as weighted means ± SD or percentages (%).

*BMI* body mass index, *eGFR* estimated glomerular filtration rate, *HDL-C* high-density lipoprotein cholesterol, *BMD* bone mineral density, *TBS* trabecular bone score, Drinking status (Had at least 12 alcohol drinks a year?).

### Univariate analysis

In weighted univariate analysis (Supplementary Table S3), some variables have a significant correlation with total TBS, including age, women, marital status, more than high school, weight, height, BMI, income to poverty ratio (High), non-drinkers, sleep, waist circumference, calcium supplementation, serum creatinine, eGFR, ALT, AST, ALP, serum albumin, blood urea nitrogen, serum calcium, cholesterol, serum phosphorus, CRP, total femur BMD, total spine BMD, and SUA. However, no significant correlation was found in the remaining variables.

### Association between SUA and total TBS

As shown in Table 2, significant negative correlations were observed between SUA and total TBS in all three multivariate linear regression models: Model 1 (β = − 0.0247, 95% CI − 0.0273, − 0.0222); Model 2 (β = − 0.0257, 95% CI − 0.0284, − 0.0230); Model 3 (β = − 0.0036, 95% CI − 0.0057, − 0.0015). In the fully adjusted model (Model 3), for every additional unit of SUA, TBS decreases by 0.0036. After converting SUA to categorical variable (quartile), the SUA of the highest quartile was 0.0120 mg/dL lower than the lowest quartile. At the same time, P for trend test all had P < 0.001, indicating that the downward trend of TBS was significant with the increase of SUA level.

**Table 2:** Association of SUA with total TBS.

	Model 1 β (95% CI) P-value	Model 2 β (95% CI) P-value	Model 3 β (95% CI) P-value
SUA per 1 mg/dL increase	− 0.0247 (− 0.0273, − 0.0222) < 0.000001	− 0.0257 (− 0.0284, − 0.0230) < 0.000001	− 0.0036 (− 0.0057, − 0.0015) 0.000644
SUA (quartile)			
Q1 (0.5–4.4 mg/dL)	Ref.	Ref.	Ref.
Q2 (4.5–5.3 mg/dL)	− 0.0179 (− 0.0275, − 0.0082) 0.000275	− 0.0158 (− 0.0245, − 0.0070) 0.000396	0.0043 (− 0.0017, 0.0103) 0.160430
Q3 (5.4–6.2 mg/dL)	− 0.0498 (− 0.0595, − 0.0402) < 0.000001	− 0.0491 (− 0.0585, − 0.0397) < 0.000001	0.0024 (− 0.0043, 0.0091) 0.484101
Q4 (6.3–11 mg/dL)	− 0.0796 (− 0.0890, − 0.0702) < 0.000001	− 0.0813 (− 0.0910, − 0.0715) < 0.000001	− 0.0120 (− 0.0174, − 0.0029) 0.005929
p for trend	< 0.001	< 0.001	< 0.001

Model 1: no covariates were adjusted. Model 2: age, gender, and race/ethnicity were adjusted. Model 3: age, gender, race/ethnicity, education, marital status, income to poverty ratio, weight, height, BMI, waist circumference, calcium supplementation, drinking status, smoke, sleep, serum creatinine, eGFR, serum albumin, alanine aminotransferase, aspartate aminotransferase, alkaline phosphatase, blood urea nitrogen, serum calcium, cholesterol, serum phosphorus, total protein, C-reactive protein, HDL-C, total femur BMD, total spine BMD.

**Figure 2:**
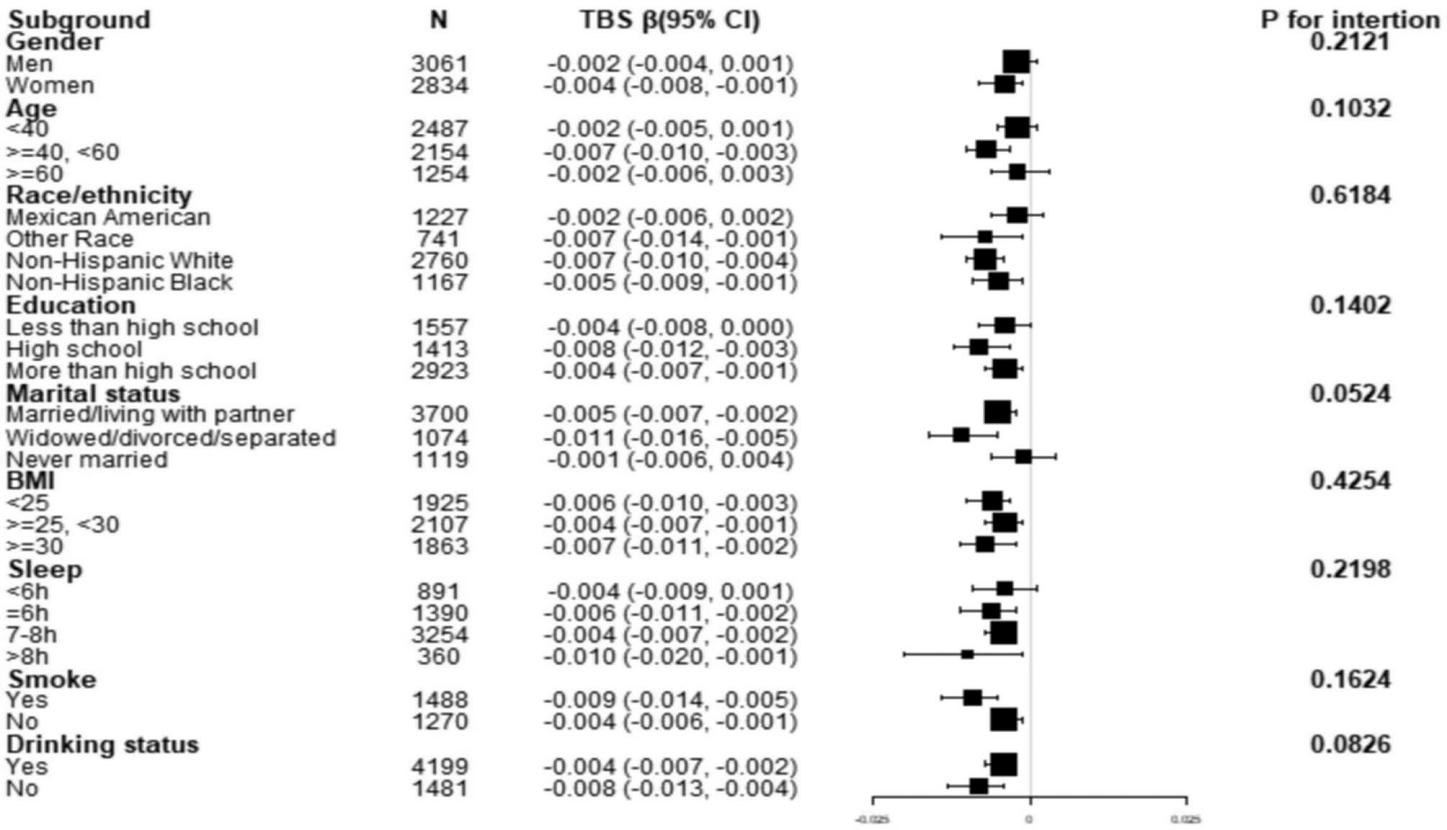
Association between SUA and total TBS according to subgroup. The model adjusts all variables except SUA, total TBS, and stratification variables.

Figure 2 reflects the results of subgroup analysis and the interaction analysis of the association between SUA and total TBS. In all subgroup analyses, there was no significant correlation between SUA and total TBS among men, adults ≥ 60 years or < 40 years, Mexican Americans, never married, sleep duration (< 6 h), and less than high school. Simultaneously, there was no interaction observed in any of the subgroups (P for interaction > 0.05).

**Figure 3:**
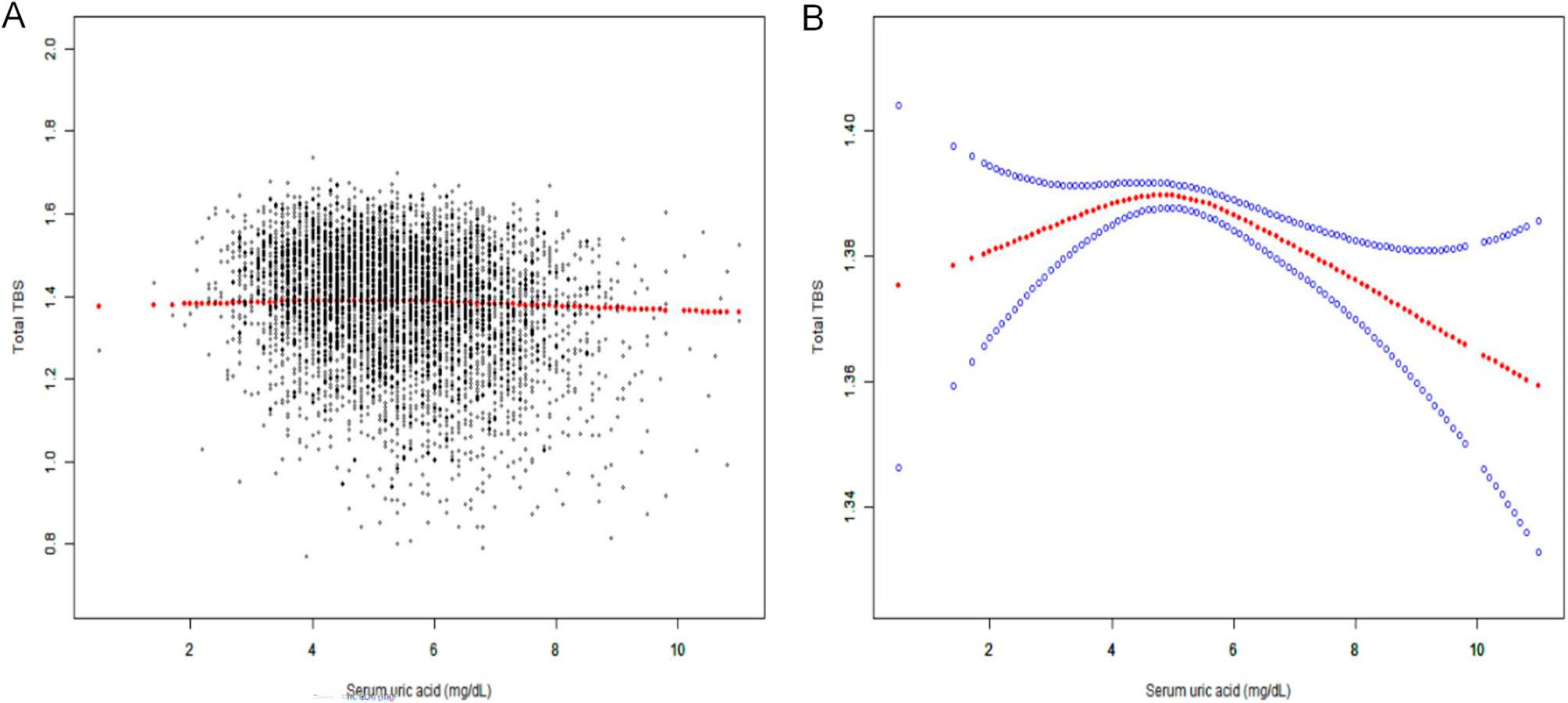
The association between SUA and total TBS. (**A**) Each black point represents a samples. (**B**) The red line is the fitted curve, and the blue bands is the 95% CI. The model adjusts all variables except SUA and total TBS.

**Figure 4:**
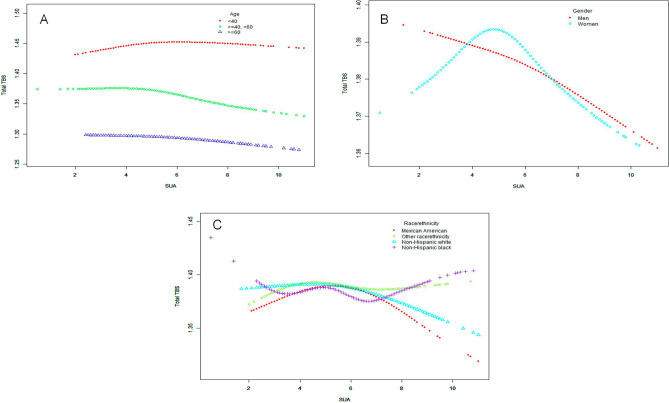
The association between SUA and total TBS stratified by age (**A**), gender (**B**) and race/ethnicity (**C**). This model adjusted for all variables except SUA, total TBS, and corresponding stratified variables.

In generalized additive models and smooth curve fittings, the nonlinear relationship and saturation effect between SUA and total TBS have been found (Fig. 3). In addition, we also found that the saturation effect value between the SUA and total TBS was 4.8 mg/dL by using a two-piecewise linear regression model (Table 3). For a SUA < 4.8 mg/dL, every 1 mg/dL increase in SUA was associated with a 0.003 greater total TBS (95% CI − 0.004–0.031, p = 0.1779). By contrast, for participants with a SUA > 4.8 mg/dL, a 1 mg/dL increase in SUA was associated with a decrease of 0.006 in total TBS (95% CI − 0.009 to − 0.004, p < 0.0001), which was statistically significant. We also examined the relationship between SUA levels and total TBS stratified by gender, age, and race/ethnicity (Fig. 4). Among women, Mexican Americans and non-Hispanic white, the relationship between SUA and total TBS was an inverted U-shaped curve, with inflection points of 4.3 mg/dL, 5.9 mg/dL and 5.7 mg/dL, respectively (Supplementary Tables S4–S6). And there are multiple inflection points among non-Hispanic black, at 4.9 mg/dL and 6.9 mg/dL (Supplementary Table S7). However, in stratified analysis, there was no statistical significance among the Mexican American population.

**Table 3:** Threshold effect analysis of SUA on total TBS by using the two-piecewise linear regression model.

Total trabecular bone score	Adjusted β (95% CI), P-value
Serum uric acid	
Fitting by the standard linear model	− 0.004 (− 0.006, − 0.002) 0.0003
Fitting by the two-piecewise linear model	
Inflection point	4.8
Serum uric acid < 4.8 (mg/dL)	0.003 (− 0.002, 0.008) 0.1779
Serum uric acid > 4.8 (mg/dL)	− 0.006 (− 0.009, − 0.004) < 0.0001
Log likelihood ratio	0.002

The model adjusts all variables except SUA and total TBS.

Models adjusted for age, gender, race/ethnicity, education, marital status, income to poverty ratio, weight, height, BMI, waist circumference, calcium supplementation, drinking status, smoke, sleep, serum creatinine, eGFR, serum albumin, alanine aminotransferase, aspartate aminotransferase, alkaline phosphatase, blood urea nitrogen, serum calcium, cholesterol, serum phosphorus, total protein, C-reactive protein, HDL-C, total femur BMD, total spine BMD.

## Discussion

In this cross-sectional study, nationally representative data from the US population aged 20 and above from 2015 to 2018 were used to evaluate the association between SUA levels and total TBS. The results showed a significant negative correlation between SUA and total TBS. The association between SUA and total TBS was not significant in men, adults aged ≥ 60 or < 40, Mexican Americans, never married, sleep duration (< 6 h), and less than high school. Additionally, we also observed a nonlinear relationship between SUA and total TBS, presenting an inverted U-shaped inflection point at 4.8 mg/dL. This inverted U-shaped association also exists among women and non-Hispanic white populations. From this, it can be inferred that controlling SUA within a reasonable range can achieve better TBS, which may help prevent osteoporosis and osteoporotic fractures.

With the rapid development of aging population, osteoporosis has become one of the most common threats to the safety of public healthcare. The final result often leads to many adverse consequences such as fractures. As is well known, BMD measurement based on DXA is an important method for diagnosing osteoporosis. However, it can only reflect bone content data and cannot provide information on bone quality^21^. For most patients with brittle fractures, the T-value is often within the range of low or even normal bone mass. In contrast, the TBS is a novel method for assessing skeletal microstructure from two-dimensional DXA images, which correlates directly with the mechanical strength of the bone^22,23^. Compared to BMD, TBS can be a tool for obtaining more comprehensive skeletal data, helping to detect individuals with microstructure degradation but normal bone density^4,22^. Higher TBS results correspond to better bone structure, while lower TBS results reflect the worse bone structure and higher fracture risk^24^. Research shows that the combination of the Fracture Risk Assessment Tool (FRAX) score and TBS can enhance the accuracy of fracture risk prediction^25,26^. Furthermore, the ability of TBS to predict fracture was not affected by BMD and most clinical risk factors^5,27^. Reviewing recent studies, numerous factors have been shown to be associated with TBS, such as obesity, sleep duration, and diabetes^28–30^. Therefore, more and more people pay attention to the related research of TBS. However, the association between serum SUA and TBS is still unclear.

Recent research reports suggest that SUA may play a beneficial role in certain diseases, such as osteoporosis^31^. This may be related to the antioxidant effect of uric acid, which can prevent oxidative stress (OS) related bone loss and osteoporosis^32^. OS can alter the process of bone remodeling by affecting the activity of osteoclasts and osteoblasts, and increase bone turnover rate, ultimately leading to osteoporosis^14,33,34^. Additionally, OS affects the proliferation of bone marrow mesenchymal stromal cells and osteoblast precursors^35,36^. Research datas show that natural antioxidants can prevent or reverse the negative effects of OS on the bone tissue by maintaining bone cell activity, activating osteoblast differentiation, and mineralization processes^37,38^. In a large cross-sectional study, a positive correlation was found between higher SUA and greater BMD^39,40^, which was supported by other Asian studies^41,42^. Similarly, a study of 17,329 participants from South Korea revealed that hyperuricemia was linked to a decreased risk of osteoporosis^43^. In addition, SUA also has the effect of preventing free radicals from damaging blood vessels, heart, and neurons^12,44^. However, the antioxidant properties of SUA may be influenced by the hydrophobic lipid layer of the cell membrane^45^. Meanwhile, SUA degradation may also produce intracellular free oxygen radicals and interact with nicotinamide adenine dinucleotide phosphate (NADPH) oxidase to enhance intracellular superoxide^46^. This result will inhibit osteoblast bone formation and stimulate osteoclast bone absorption.

Although SUA may have contradictory effects on bone metabolism, our study further indicates a significant negative correlation between SUA levels and total TBS. Whether the covariates are adjusted or not, this result is statistically significant. In the subgroup analysis, this negative correlation still exists, especially among women aged 40 to 60 (≥ 40, < 60) with a high school education or above, excluding Mexican Americans. We also found that there is a generally lower total TBS value in elderly patients (≥ 60). Maintaining uric acid at an appropriate level to obtain a high total TBS value helps to further prevent the occurrence of osteoporotic fractures and provides guidance for clinical practice.

In this study, some advantages are worth noting. First of all, this is the first study to explore the correlation between SUA levels and total TBS. Next, this study used large sample data and a multivariate adjusted model to control for related confounding factors. In addition, we conducted stratified analysis, generalized additive model, and smooth curve fitting analysis to discover the multifaceted effects of SUA levels on total TBS. Of course, this study also has its limitations. Firstly, this study is a cross-sectional study and cannot determine the causal relationship between SUA levels on total TBS. Secondly, the research subjects are mainly American participants, and it is not yet known whether they are applicable to other regions or countries. Furthermore, variations in dietary and lifestyle habits may also influence uric acid levels^47,48^.

## Conclusion

In the adult population of the US, our study found a significant negative correlation between SUA levels and total TBS, which follows an inverted U-shaped curve (inflection point: 4.8 mg/dL). Meanwhile, this inverted U-shaped curve also exists among non-Hispanic white women. This study suggests that maintaining SUA at a saturation level can provide the optimal total TBS value for adults and may help prevent osteoporosis and osteoporotic fractures.

### Supplementary Information

Supplementary Tables.

## Data Availability

The data that support the results of this study can obtained from https://wwwn.cdc.gov/nchs/nhanes/Default.aspx. The availability of this data is not restricted.
