# Supplementary material for: Cross-sectional analysis of the correlation between serum uric acid and trabecular bone score: NHANES 2005–2008
Source: Sci Rep. 2023 Dec 6;13:21546. doi: 10.1038/s41598-023-48739-5 (PMC10700542; doi:10.1038/s41598-023-48739-5)
Supplement: Supplementary file 1 — Supplementary Tables. [file 41598_2023_48739_MOESM1_ESM.docx]

**Supplementary Table S1.** The equation for estimating GFR based on CKD-EPI.

| **Race and gender** | **Serum Creatinine mg/dL** | **Equation** |
| --- | --- | --- |
| Black |  |  |
| Female | ≤0.7 | GFR = 166 × (Scr/0.7)-0.329 × (0.993)Age |
|  | >0.7 | GFR = 166 × (Scr/0.7)-1.209 × (0.993)Age |
| Male | ≤0.9 | GFR = 163 × (Scr/0.9)-0.411 × (0.993)Age |
|  | >0.9 | GFR = 163 × (Scr/0.9)-1.209 × (0.993)Age |
| White or other |  |  |
| Female | 0.7 | GFR = 144 × (Scr/0.7)-0.329 × (0.993)Age |
|  | >0.7 | GFR = 144 × (Scr/0.7)-1.209 × (0.993)Age |
| Male | ≤0.9 | GFR = 141 × (Scr/0.9)-0.411 × (0.993)Age |
|  | >0.9 | GFR = 141 × (Scr/0.9)-1.209 × (0.993)Age |

Abbreviation: GFR (glomerular filtration rate), CKD-EPI (Chronic Kidney Disease Epidemiology Collaboration)

**Supplementary Table S2.** Description of the missing variables.

| **Variables** | **No** | **Missing data, N** |
| --- | --- | --- |
| Gender | 5895 | 0 |
| Age | 5895 | 0 |
| Race/Ethnicity | 5895 | 0 |
| Education | 5895 | 2 |
| Marital.Status | 5895 | 2 |
| Income to poverty ratio | 5895 | 0 |
| Weight | 5895 | 0 |
| Height | 5895 | 0 |
| BMI | 5895 | 1 |
| Drinking status | 5895 | 215 |
| Sleep | 5895 | 0 |
| Smoke | 5895 | 3137 |
| Waist circumference | 5895 | 4 |
| Calcium supplementation | 5895 | 719 |
| Serum creatinine | 5895 | 40 |
| eGFR | 5895 | 0 |
| Albumin | 5895 | 0 |
| Alanine aminotransferase | 5895 | 40 |
| Aspartate aminotransferase | 5895 | 40 |
| Alkaline phosphatase | 5895 | 79 |
| Blood urea nitrogen | 5895 | 0 |
| Total calcium | 5895 | 0 |
| Cholesterol | 5895 | 1 |
| Phosphorus | 5895 | 42 |
| Total protein | 5895 | 14 |
| C-reactive protein | 5895 | 45 |
| HDL-C | 5895 | 136 |
| Total femur BMD | 5895 | 285 |
| Total spine BMD | 5895 | 611 |
| Total TBS | 5895 | 0 |
| Serum uric acid (mg/dL) | 5895 | 0 |

Abbreviation: BMI (body mass index), eGFR (estimated glomerular filtration rate), HDL-C (high-density lipoprotein cholesterol), BMD (bone mineral density), Drinking status (Had at least 12 alcohol drinks a year?), TBS (trabecular bone score).

**Supplementary Table S3**. The results of univariate analysis, weighted.

|  | **Statistics** | **Total TBS β(95% CI)** | **P-value** |
| --- | --- | --- | --- |
| Gengder (%) |  |  |  |
| Men | 3061 (51.93%) | Ref |  |
| Women | 2834 (48.07%) | 0.02 (0.01, 0.03) | <0.0001 |
| Age (years) | 44.66 ± 16.00 | -0.00 (-0.00, -0.00) | <0.0001 |
| Income to poverty ratio (%) |  |  |  |
| Low | 2348(39.83) | Ref |  |
| Middle | 1577(26.75) | 0.02 (0.01, 0.03) | 0.068 |
| High | 1970(33.42) | -0.00 (-0.00, -0.00) | <0.0001 |
| Race/ethnicity (%) |  |  |  |
| Mexican American | 1227 (20.81%) | Ref |  |
| Other race/ethnicity | 741 (12.57%) | 0.01 (-0.01, 0.02) | 0.3790 |
| Non-Hispanic white | 2760 (46.82%) | 0.00 (-0.01, 0.01) | 0.8025 |
| Non-Hispanic black | 1167 (19.80%) | -0.01 (-0.02, 0.01) | 0.2845 |
| Education (%) |  |  |  |
| Less than high school | 1557 (26.41%) | Ref |  |
| High school | 1413 (23.97%) | 0.00 (-0.01, 0.01) | 0.5894 |
| More than high school | 2923 (49.58%) | 0.04 (0.03, 0.05) | <0.0001 |
| Not recorded | 2 (0.03%) | -0.07 (-0.22, 0.08) | 0.3615 |
| Marital status (%) |  |  |  |
| Married/living with partner | 3700 (62.77%) | Ref |  |
| Widowed/divorced/separated | 1074 (18.22%) | -0.04 (-0.05, -0.03) | <0.0001 |
| Never married | 1119 (18.98%) | 0.05 (0.04, 0.06) | <0.0001 |
| Not recorded | 2 (0.03%) | -0.08 (-0.23, 0.07) | 0.3043 |
| Income to poverty ratio | 2.70 ± 1.58 | 0.00 (0.00, 0.01) | 0.0051 |
| Weight (kg) | 79.48 ± 17.87 | -0.00 (-0.00, -0.00) | <0.0001 |
| Height (cm) | 168.22 ± 9.99 | 0.00 (0.00, 0.00) | 0.0105 |
| BMI (kg/m^2^) | 28.04 ± 5.69 | -0.01 (-0.01, -0.01) | <0.0001 |
| Drinking status (%) |  |  |  |
| Yes | 4199 (71.23%) | Ref |  |
| No | 1481 (25.12%) | -0.02 (-0.03, -0.01) | <0.0001 |
| Not recorded | 215 (3.64%) | -0.00 (-0.02, 0.02) | 0.8975 |
| Sleep (%) |  |  |  |
| <6h | 891 (15.11%) | Ref |  |
| =6h | 1390 (23.58%) | 0.02 (0.01, 0.03) | 0.0016 |
| 7-8h | 3254 (55.20%) | 0.03 (0.02, 0.04) | <0.0001 |
| >8h | 360 (6.11%) | 0.02 (0.00, 0.04) | 0.0381 |
| Smoke (%) |  |  |  |
| Yes | 1488 (25.24%) | Ref |  |
| No | 1270 (21.54%) | -0.03 (-0.04, -0.02) | <0.0001 |
| Not recorded | 3137 (53.22%) | -0.00 (-0.00, 0.00) | 0.8758 |
| Waist circumference (cm) | 96.02 ± 14.21 | -0.01 (-0.01, -0.00) | 0.0139 |
| Calcium supplementation (mg) | 892.43 ± 551.98 | 0.00 (0.00, 0.00) | <0.0001 |
| Serum creatinine (mg/dL) | 0.86 ± 0.18 | -0.02 (-0.04, -0.00) | 0.0283 |
| eGFR (mL/min/1.73 m2) | 98.62 ± 18.92 | 0.00 (0.00, 0.00) | <0.0001 |
| Serum albumin (g/L) | 42.60 ± 3.13 | 0.01 (0.01, 0.01) | <0.0001 |
| Alanine aminotransferase (U/L) | 26.73 ± 21.45 | -0.00 (-0.00, -0.00) | <0.0001 |
| Aspartate aminotransferase (U/L) | 26.27 ± 18.10 | -0.00 (-0.00, -0.00) | <0.0001 |
| Alkaline phosphatase (U/L) | 69.26 ± 22.45 | -0.00 (-0.00, -0.00) | <0.0001 |
| Blood urea nitrogen(mg/dL) | 11.90 ± 3.87 | -0.00 (-0.00, -0.00) | <0.0001 |
| Serum calcium (mg/dL) | 9.45 ± 0.36 | 0.01 (0.00, 0.02) | 0.0314 |
| Cholesterol (mg/dL) | 199.73 ± 40.60 | -0.00 (-0.00, -0.00) | <0.0001 |
| Serum phosphorus (mg/dL) | 3.76 ± 0.56 | 0.01 (-0.00, 0.01) | 0.1007 |
| Total protein (g/L) | 71.90 ± 4.54 | 0.00 (-0.00, 0.00) | 0.5307 |
| C-reactive protein (mg/L) | 0.37 ± 0.73 | -0.03 (-0.04, -0.03) | <0.0001 |
| HDL-C (mmol/L) | 1.37 ± 0.41 | 0.05 (0.05, 0.06) | <0.0001 |
| Total femur BMD(g/cm^2^) | 0.99 ± 0.15 | 0.14 (0.12, 0.16) | <0.0001 |
| Total spine BMD (g/cm^2^) | 1.04 ± 0.14 | 0.33 (0.30, 0.35) | <0.0001 |
| SUA (mg/dL) | 5.36 ± 1.33 | -0.02 (-0.03, -0.02) | <0.0001 |

Data are expressed as weighted means ± SD or percentages (%).

Abbreviation: CI (confidence interval), BMI (body mass index), eGFR (estimated glomerular filtration rate), HDL-C (high-density lipoprotein cholesterol), BMD (bone mineral density), TBS (trabecular bone score), Drinking status (Had at least 12 alcohol drinks a year?), SUA (Serum uric acid), Ref (reference).

**Supplementary Table S4.** Threshold effect analysis of SUA on total TBS in women using the two-piecewise linear regression model. The model adjusts all variables except SUA, total TBS, and gender.

| **Total trabecular bone score** | **Adjusted β (95% CI), P-value** |
| --- | --- |
| Gender for Women |  |
| Fitting by the standard linear model | -0.005 (-0.008, -0.002) 0.0011 |
| Fitting by the two-piecewise linear model |  |
| Inflection point | 4.3 |
| Serum uric acid <4.3 (mg/dL) | 0.006 (-0.002, 0.013) 0.1604 |
| Serum uric acid >4.3 (mg/dL) | -0.009 (-0.014, -0.005) <0.0001 |
| Log likelihood ratio | 0.002 |

**Supplementary Table S5.** Threshold effect analysis of SUA on total TBS stratified by age. The model adjusts all variables except SUA, total TBS, and age.

| **Total trabecular bone score** | **Age adjusted β (95% CI), P-value** | | |
| --- | --- | --- | --- |
|  | **<40** | **>=40, <60** | **>=60** |
| **Fitting by the standard**  **linear model** | -0.002 (-0.006, -0.000) 0.0081 | -0.006 (-0.010, -0.003) 0.0006 | -0.004 (-0.008, 0.001) 0.1159 |
| **Fitting by the two-piecewise linear model** | |  |  |
| **Inflection point (K)** | 4.7 | 3.9 | 7.6 |
| < K, effect 1 | 0.007 (-0.001, 0.014) 0.0715 | 0.008 (-0.000, 0.033) 0.0528 | -0.002 (-0.007, 0.003) 0.4949 |
| > K, effect 2 | -0.006 (-0.009, -0.002) 0.0026 | -0.008 (-0.012, -0.004) <0.0001 | -0.025 (-0.048, -0.002) 0.0350 |
| **Log likelihood ratio** | 0.064 | 0.056 | 0.062 |

**Supplementary Table S6.** Threshold effect analysis of SUA on total TBS stratified by race/ethnicity. The model adjusts all variables except SUA, total TBS, and race/ethnicity.

| **Total trabecular bone score** | **Race/ethnicity adjusted β (95% CI), P-value** | | |
| --- | --- | --- | --- |
|  | **Mexican American** | **Other race/ethnicity** | **Non-Hispanic white** |
| Fitting by the standard  linear model | 0.002 (-0.002, 0.006) 0.3465 | -0.006 (-0.012, 0.000) 0.0716 | -0.004 (-0.007, -0.001) 0.0068 |
| Fitting by the two-piecewise linear model | | | |
| Inflection point (K) | 5.9 | 6.4 | 5.7 |
| < K, effect 1 | 0.013 (0.007, 0.020) <0.0001 | -0.012 (-0.020, -0.003) 0.0071 | 0.001 (-0.004, 0.006) 0.6926 |
| > K, effect 2 | -0.013 (-0.021, -0.005) 0.0013 | 0.007 (-0.007, 0.021) 0.3254 | -0.009 (-0.014, -0.004) 0.0002 |
| Log likelihood ratio | <0.001 | 0.052 | 0.009 |

**Supplementary Table S7.** Threshold effect analysis of SUA on total TBS stratified by race/ethnicity. The model adjusts all variables except SUA, total TBS, and race/ethnicity.

| **Total trabecular bone score** | **Race/ethnicity adjusted β (95% CI), P-value** |
| --- | --- |
|  | **Non-Hispanic black** |
| Fitting by the standard  linear model | -0.003 (-0.007, 0.002) 0.1989 |
| Fitting by the two-piecewise linear model | |
| Inflection point (K1, K2) | 4.9, 6.9 |
| < K1, effect 1 | 0.001 (-0.009, 0.012) 0.7759 |
| K1-K2, effect 2 | -0.015 (-0.025, -0.005) 0.0032 |
| > K2, effect 3 | 0.010 (-0.007, 0.027) 0.2685 |
| Log likelihood ratio | 0.002 |
